# Antimicrobial resistance detection in Southeast Asian hospitals is critically important from both patient and societal perspectives, but what is its cost?

**DOI:** 10.1371/journal.pgph.0000018

**Published:** 2021-10-13

**Authors:** Tamalee Roberts, Nantasit Luangasanatip, Clare L. Ling, Jill Hopkins, Risara Jaksuwan, Yoel Lubell, Manivanh Vongsouvath, H. Rogier van Doorn, Elizabeth A. Ashley, Paul Turner

**Affiliations:** 1 Lao-Oxford-Mahosot Hospital- Wellcome Trust Research Unit, Mahosot Hospital, Vientiane, Lao People’s Democratic Republic; 2 Nuffield Department of Medicine, Centre for Tropical Medicine and Global Health, University of Oxford, Oxford, United Kingdom; 3 Faculty of Tropical Medicine, Mahidol-Oxford Tropical Medicine Research Unit, Mahidol University, Bangkok, Thailand; 4 Shoklo Malaria Research Unit, Mahidol-Oxford Tropical Medicine Research Unit, Mae Sot, Thailand; 5 Cambodia Oxford Medical Research Unit, Angkor Hospital for Children, Siem Reap, Cambodia; 6 Oxford University Clinical Research Unit, National Hospital for Tropical Diseases, Hanoi, Vietnam

## Abstract

Antimicrobial resistance (AMR) is a major threat to global health. Improving laboratory capacity for AMR detection is critically important for patient health outcomes and population level surveillance. We aimed to estimate the financial cost of setting up and running a microbiology laboratory for organism identification and antimicrobial susceptibility testing as part of an AMR surveillance programme. Financial costs for setting up and running a microbiology laboratory were estimated using a top-down approach based on resource and cost data obtained from three clinical laboratories in the Mahidol Oxford Tropical Medicine Research Unit network. Costs were calculated for twelve scenarios, considering three levels of automation, with equipment sourced from either of the two leading manufacturers, and at low and high specimen throughput. To inform the costs of detection of AMR in existing labs, the unit cost per specimen and per isolate were also calculated using a micro-costing approach. Establishing a laboratory with the capacity to process 10,000 specimens per year ranged from $254,000 to $660,000 while the cost for a laboratory processing 100,000 specimens ranged from $394,000 to $887,000. Excluding capital costs to set up the laboratory, the cost per specimen ranged from $22–31 (10,000 specimens) and $11–12 (100,000 specimens). The cost per isolate ranged from $215–304 (10,000 specimens) and $105–122 (100,000 specimens). This study provides a conservative estimate of the costs for setting up and running a microbiology laboratory for AMR surveillance from a healthcare provider perspective. In the absence of donor support, these costs may be prohibitive in many low- and middle- income country (LMIC) settings. With the increased focus on AMR detection and surveillance, the high laboratory costs highlight the need for more focus on developing cheaper and cost-effective equipment and reagents so that laboratories in LMICs have the potential to improve laboratory capacity and participate in AMR surveillance.

## Introduction

Antimicrobial resistance (AMR) is a major threat to public health. Drug resistant infections can lead to extended hospital stays and higher hospital costs. The hospital cost for treating a resistant organism has been estimated to be $10,000 to $40,000 higher than treating a susceptible organism in Europe [1]. Overall, there has been little research to date on the cost of AMR in low- and middle- income countries (LMICs) [2], but the total economic cost of AMR due to resistance in five pathogens (*Staphylococcus aureus, Escherichia coli, Klebsiella pneumoniae, Acinetobacter baumannii* and *Pseudomonas aeruginosa*) has been estimated at $0.5 billion in Thailand [3]. These high costs are not only of consequence to the health care provider, but often the individual patient will have to bear the high costs. Though the drivers of AMR are multifactorial, the use of antibiotics is the biggest driver of AMR with overuse and incorrect prescriptions playing a large role in this [4]. Where possible, patients should have specimens taken for testing to determine the infecting organism/s and antibiotic susceptibility profiles, to inform treatment. Laboratory diagnosis is also key for AMR surveillance with proportions of specific bug-drug susceptibility informing the level of resistance seen in a community. However, for the testing of specimens and AMR surveillance, there needs to be laboratories available with sufficient capacity to provide reliable results.

A recent report showed that laboratory surveillance and reference costs constitute 67–77% of total AMR surveillance costs, depending on the level of surveillance [5]. However, the actual cost for laboratory processing of samples for AMR detection have received little attention, especially in LMICs. The WHO Global Antimicrobial Resistance Surveillance System (GLASS) Early Implementation report (2020) stated that there were higher rates of resistant bacteria in LMICs than in the upper-middle and high-income countries, though selection bias may play a role [6]. As noted above, laboratory capacity is crucial for AMR surveillance, however, capacity for such surveillance in LMICs varies. Overall, there is limited standardized and comprehensive AMR data from Southeast Asia and the Western Pacific with only 7/27 countries in the Western Pacific region reporting data to GLASS in 2019 [6]. The WHO has also described the AMR problem in Southeast Asia as “neglected” [7,8]. Importantly, it is not possible to set up a laboratory for AMR detection, without also setting up all other functional components including safety and quality control. Procedures need to be in place for initial specimen processing, organism detection and identification (ID) before antimicrobial susceptibility testing (AST).

There is increasing emphasis on laboratory capacity strengthening for AMR surveillance, with six country Fleming Fund grants in process in the Western Pacific/ Southeast Asia regions (Indonesia, Laos, Myanmar, Papua New Guinea, Timor-Leste and Vietnam) and a total of 24 country grants in Asia and Africa with a value of £265 million (www.flemingfund.org). However, there is little information available on the cost of setting up and running these laboratories. Therefore, the aim of this study was to estimate the cost of setting up (infrastructure, equipment and personal protective equipment) and running (general consumables, media, antibiotic discs, equipment maintenance, staff and utilities) a microbiology laboratory for organism identification and susceptibility testing as part of the AMR surveillance programme in the Southeast Asia region, to support policy decision makers considering additional investment in setting up laboratories for national AMR surveillance.

## Materials and methods

### Ethics statement

There were no human participants involved in this study and therefore ethics was not required.

### Approach outline

Our first objective was to estimate the financial cost to set up a microbiology laboratory where no such facilities exist as well as the annual running cost in a hospital setting over a five-year time period. For this, we took a ‘top-down’, comprehensive assessment of establishing and maintaining a laboratory where no such capacity was in place, therefore including capital, labour and consumables. We considered the costs of establishing laboratories of varying capacities in a Southeast Asian setting and their routine running cost at different levels of specimen throughput, as detailed below. This approach is useful for generating broader costs for setting up and running the laboratories, and average costs per specimen; its shortcoming however is that it cannot provide more detailed costing for specific specimen types or activities. Our second objective was to cost specifically the activities associated with AMR surveillance for high priority organisms, which would also be informative for existing laboratories where AMR surveillance activities might be expanded. For this, we carried out a micro-costing exercise for the cost per specimen and isolates for the subset of high priority pathogens, capturing consumables alone, which will be of most relevance where laboratory capacity is in place, to inform the costs for expanding AMR surveillance activities. Unit costs for the resources used were derived using market value. Discounting was not applied as this would not be relevant for financial costing and planning, and no adjustment was made for inflation as the resource data and unit costs were all recent, therefore the impact would be negligible.

### Total cost of setting up a microbiology laboratory (top down approach)

We generated a total of 12 cost scenarios, comprised of two levels of specimen throughput (reflecting a low capacity laboratory with a throughput of 10,000 specimens per year, and a medium/high capacity with a throughput of 100,000 specimens per year); three levels of automation, with the relevant equipment sourced from two instrument manufacturers: i) automated blood culture system (BACTEC or BacT/ALERT) with manual identification (ID) and antimicrobial susceptibility testing (AST), ii) automated blood culture and AST (BACTEC + Phoenix or BacT/ALERT + Vitek AST), and iii) automated blood culture, ID and AST (BACTEC + Bruker MALDI-TOF + Phoenix, or BacT/ALERT + Vitek MS MAL-DI-TOF + Vitek AST). Instruments were grouped by manufacturer to reflect likely cost savings when purchased together (Becton Dickinson, New Jersey, USA; Bruker Daltonik GmbH, Bremen, Germany and bioMérieux, Marcy-l’Étoile, France), acknowledging, however, that in reality a combination of the different company products is often found in laboratories.

### Unit cost of surveillance testing (micro-costing approach)

**Fig 1:**
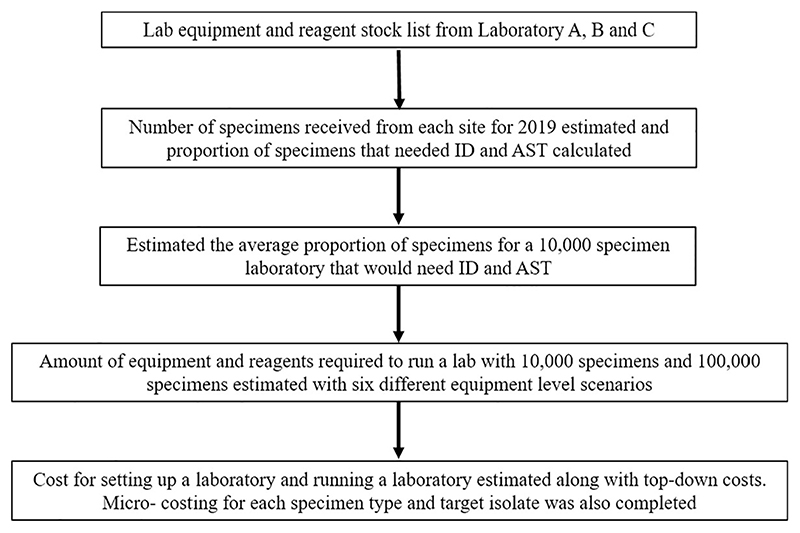
Flowchart of methods used to estimate the cost of setting up and running a microbiology laboratory in Southeast Asia.

A micro-costing approach was used to estimate the cost per specimen (blood culture, cerebrospinal fluid (CSF), sputum and urine) and per AMR surveillance target organism (*Streptococcus pneumoniae, S. aureus, Salmonella* spp, *Escherichia coli, K. pneumoniae* and *Acinetobacter* spp) derived from the cost of specific consumables and reagents, dependent on the amount used for processing that specimen or isolate. These six target organisms were chosen due to their status of being of international concern by the WHO [9] in blood and their regional implications for AMR in both the Western Pacific and Southeast Asia [10]. This calculation did not take into account equipment, maintenance, staffing, general consumables and safety costs but focuses on the specific items for processing one specimen. Fig 1 summarises the methods used to estimate the cost of setting up and running a microbiology laboratory.

### Data sources

A list of the basic equipment, reagents and consumables needed for a microbiology laboratory was created based on current laboratory records from three clinical laboratories from the Mahidol-Oxford Tropical Medicine Research Unit (MORU) network and expert opinion from members of the MORU clinical microbiology group. These laboratories included: Laboratory A—Mahosot Hospital Microbiology Laboratory, Vientiane Capital, Lao People’s Democratic Republic (Lao PDR, Laos); Laboratory B—the Cambodia Oxford Medical Research Unit (COMRU), Siem Reap, Cambodia; and Laboratory C—Shoklo Malaria Research Unit (SMRU), Tak Province, Thailand. Laboratory A supports the diagnostic service for Mahosot Hospital, a ~400-bed government hospital providing primary, secondary, and tertiary care and admitting ~2,000 patients/month and is partly supported by the Lao- Oxford- Mahosot Hospital Wellcome Trust Research Unit (LOMWRU) [11]. The laboratory also receives specimens from hospitals within Vientiane Capital as well as several provincial hospitals. Laboratory B supports the diagnostic service at Angkor Hospital for Children, a ~100-bed non-governmental paediatric hospital in Siem Reap [12]. Laboratory C supports several clinics located along the Thailand-Myanmar border that provide outpatient and inpatient healthcare to marginalised populations [13]. The individual cost of each piece of equipment, reagent and consumable was obtained from recent quotes from any of the three laboratories or, if not available, new quotes were requested from companies directly. Where there was a large discrepancy in the price of the same item for different laboratories, the average cost was calculated and used. Reflecting the location of the MORU logistics hub in Bangkok, the quotes were predominantly in Thai Baht and from local distributors. Set up costs comprised of equipment and supplies, office space, safety equipment, and building while the running costs were consumable items, reagents (including agar plates, biochemicals and identification test kits), antibiotic discs and Etests, quality assurance, human resources, electricity, and maintenance costs. Both direct and indirect costs were included to estimate the total cost over a five-year running period. The costs were tabulated and calculated using Microsoft Excel (Redmond, WA, USA). Costs were converted to US Dollars ($, 2020) with the exchange rate $1 = 31.16 Thai Baht and 1GBP = 40.09 Thai Baht [14]. Specimen numbers from the year 2019 from the three clinical laboratories were used to estimate the characteristics of specimens processed in a laboratory receiving 10,000 and 100,000 specimens in a year and the number of each item required to test for those specimens. Construction costs were estimated from a Kenyan laboratory from a previous report [5] and utility costs were estimated from Laboratory B and C.

## Results

### Top down approach

Based on the number of specimen data processed from all three laboratories in 2019 (Table 1), it was estimated that 50% of specimens received are blood cultures, 20% are urine, 2% are CSF, 2% are sputum/endotracheal aspirates and 26% are a mixture of other specimen types.

**Fig 2:**
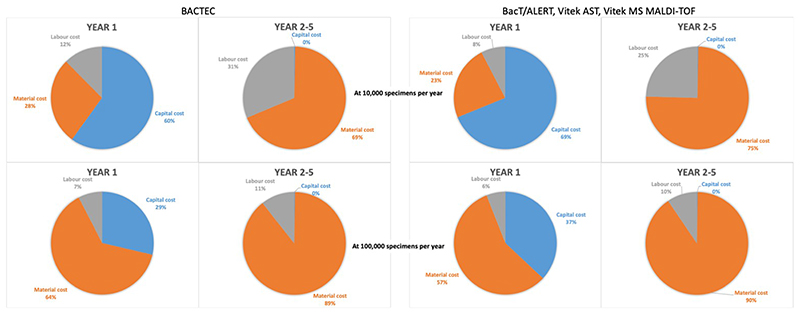
Contribution of the cost of setting up a microbiology laboratory using the BACTEC (lowest cost) and using the BacT/Alert, Vitek AST, Vitek MS MALDI-TOF (highest cost) at two scales (10,000 specimens per year and 100,000 specimens per year) from top down approach. Capital costs include equipment purchasing, labour costs include staff wages and material costs include consumables, reagents and equipment maintenance.

Approximately 5% of blood cultures, 10% of urines, 5% of CSF specimens and 50% of sputum/endotracheal aspirates are culture positive and would need further work up including ID and AST. From these estimations, consumable, reagent and equipment requirements were calculated based on low specimen capacity and medium/high specimen capacity processing per year following a list of assumptions (S1 Table, S1 Materials). The total estimated costs of setting up a laboratory based on the top down approach for the three different scenarios with the two different specimen sizes are presented in Table 2. The set-up costs for a low specimen capacity laboratory ranged from $254,000 to $660,000 while the set-up cost for a medium/high specimen capacity laboratory ranged from $394,000 to $887,000. The option of a BacT/ALERT blood culture machine with no automated ID or AST had the lowest cost while the full automation option with the BACTEC, Phoenix and Bruker MALDI-TOF had the highest set up costs. In the first year, the highest cost contribution is the capital cost (60–69%) followed by material cost (23–28%) and labour cost (8–12%) with the workload of 10,000 specimens per year while material cost has the highest contribution (57–64%) followed by capital cost (29–37%) and labour cost (6–7%) at 100,000 specimens per year. In the subsequent years, the majority of cost contribution is material cost (69–90%) followed by the labour cost (10–31%) with no capital cost in both workload levels (Fig 2). Although the option with the BACTEC, Phoenix and Bruker MALDI-TOF had the highest initial set up costs, the annual running costs were lower than BacT/ALERT, Vitek AST and Vitek MS MALDI-TOF in both workload scenarios. The overall average cost per year was similar for laboratories using equipment from either of the two companies in each of the three scenarios.

The average cost per specimen based on the overall cost using the top-down approach ranged from $22 to $31 for the low throughput scenarios and from $11 to $12 for the medium/high throughput scenarios. This analysis does not, however, provide cost estimates for specific specimen types. With the top-down approach, the unit cost per isolate ranged from $215 to $304 for the low capacity specimen level and from $105 to $122 for the medium/high capacity specimen level. These estimates were based on the three sites data that approximately 10% of specimens would yield isolates that will have full work up and does not take into consideration the specific tests that are required for each different organism type.

### Micro-costing approach

Considering only test costs for the specific specimen and isolates (and not the whole scope of the laboratory equipment and processes required), the direct cost for processing a specimen ranged from $1.65 for urines to $5.26 for positive blood cultures. S2 Table shows the direct costs for processing blood cultures (positive and negative), CSF, sputum and urines. This table also shows the estimated cost for processing similar specimens from the Kenyan laboratory capacity report [5]. The cost per isolate ranged from $7.86 for *E. coli* and *K. pneumoniae* to $19.50 for *Salmonella* spp. (detailed in S3 Table). These costs would vary by laboratory depending on the precise testing procedures implemented.

## Discussion

This study provides a conservative estimate of the costs for setting up and running a microbiology laboratory for AMR surveillance in the Southeast Asia region from a healthcare provider perspective. We provide estimates for the cost of setting up and running a laboratory and also a summary of the equipment and consumables costs that are required for expanding throughput in existing laboratories. Our findings will be useful for decision makers at both the hospital and national level when contemplating resource allocation for AMR surveillance.

The estimated set-up costs for a laboratory aiming to process 10,000 specimens annually ranged from $254,000 to $660,000 using the top- down approach. This is comparable to the figure for setting up laboratories in Kenya, estimated at £353,501 ($263,600) [5]. However, the figure from Kenya did not include safety-related costs, including personal protective equipment and biological/chemical spill kits, and included substantially less equipment than in our analysis. In addition, the annual costs of running the laboratory from this study were also lower than our estimates, ~ £138,877 ($105,546) for ~6,000 specimens compared with approximately $185,000 for 10,000 specimens in our study.

From our analysis, the direct costs of processing specific specimens ranged from $1.65 for urines to $5.26 for positive blood cultures (S2 Table) whereas the indirect costs, taking into account staffing and equipment, ranged from $22 to $31 depending on equipment capacity level. The direct cost estimates can help established laboratories with budgeting for expanded AMR surveillance activities, and understand individual specimen costs whereas the indirect approach allows for the estimation of the cost for establishing and running laboratories including overheads, equipment, staff and utilities. The direct specimen costs were similar for blood culture and urine with the Kenyan laboratory analysis but differed significantly for CSF and sputum [5]. These differences highlight the importance for individual laboratories performing their own specimen specific costing as local methods, media and consumable costs will have a significant impact on the costs. Of relevance to LMIC settings is the issue that laboratories with lower specimen throughput may waste considerable quantities of reagents due to expiration: these costs may be significant and not well captured by direct cost estimation.

The per-specimen costs for setting-up and running a diagnostic microbiology laboratory are high for a LMIC, where costs of microbiology testing are often passed on to patients. Governments aiming for universal healthcare coverage are unlikely to be able to cover these costs with their current designated health expenditure. For example, in Laos the 2017 public expenditure on health from domestic sources was $22 per capita, which would not cover the cost of processing a single specimen ($22- $31) [15]. The situation is similar for Cambodia, spending $20 per capita and Myanmar at $9 per capita on health expenditure [15]. While initiatives like the Fleming Fund are invaluable in supporting the initial outlay for laboratory costs, maintaining these running costs after the initiative finishes will rely on local governments, which in many places would not be sustainable.

The high cost of equipment and reagents cannot be justified, especially in LMICs. A small number of manufacturers dominate the market for equipment and reagents currently, and there is little incentive to lower the cost to end users. There has been a lot of progress in developing new diagnostic technologies in recent years, such as automated identification and susceptibility testing, and selective media to facilitate identification of drug resistant organisms. These can improve the quality and efficiency of microbiology diagnosis, however they are usually out of reach of government laboratories in LMICs either because of cost or because they are not adapted to use in tropical conditions [16].

Importantly, the inclusion of on-going maintenance costs is critical when planning laboratory capacity development: such costs can be substantial but are frequently neglected and may lead to early equipment failure and subsequent workflow difficulties. Often medical equipment may be donated or bought as part of a grant, but the yearly maintenance costs are not included. This can have a substantial impact on budgets and affect whether equipment will be adequately maintained [17].

While this study focused on the cost of setting up and running a laboratory for AMR surveillance, many resources and costs were included that are not specifically related to AMR detection. A diagnostic laboratory must have the capacity to identify a broad range of organisms and, as the infectious agent is not known until it has been isolated on agar and had identification tests completed, all general identification tests were included in our costing exercise. These costs will vary depending on laboratory workflows/procedures, endemic diseases in countries/regions and their AMR patterns. For example, in Southeast Asia *Burkholderia pseudomallei* and *Salmonella* Typhi are endemic, so additional identification tests for those organisms are included in this analysis. However, currently multi-drug resistant (MDR) *S*. Typhi is rare in Laos [18], but common in other Asian countries [19–21].

From the perspective of AMR surveillance programme implementation, apart from the cost of setting up and running a laboratory there are many other costs that need to be taken into consideration. These include costs for training of local staff on data management and analysis, establishing and maintaining countrywide database systems, and development and dissemination of standard operating procedures. At the site level, in addition to laboratory staff, there is also a need for administration, stock management and logistics staff (procurement, budget, management). As some of these staff may be part of a greater laboratory system in the hospital, or they might be required specifically for the microbiology laboratory, we have not included these costs as they will be different for each site. However, all of these costs should be included as part of the national AMR surveillance programme budgets.

There are several additional limitations to this study. Firstly, not all the indirect costs could be accurately estimated or included. Electricity costs were estimated from two laboratories, which may not be an accurate representation for every country or the region. Building costs were based on the Kenyan laboratory capacity report and may not be a true representation for build costs in Southeast Asia. While staff salaries were included, they were a minimal estimate and could vary greatly depending on country and between sectors (private vs. public). Waste management costs were not included in the analysis, since this may be organised by the host hospital and therefore the laboratory may not incur a direct fee. Laboratory identification capacity was costed to include all major pathogen groups, with the exception of anaerobes. It is recognised that this is not ideal, but it is a frequent limitation for laboratories operating in lower resource settings. Specific costs of horse/sheep blood, a critical component of blood agar, were not included since this may often be acquired informally (e.g. from local farmers) and may not be expensive so would not add greatly to our estimations. Manual blood culture techniques were not included in the analysis due to the focus on the cost of automated equipment, but it is recognised that many laboratories in LMICs will only have resources for manual blood culture processing. While the overall cost without the blood culture machine will be greatly reduced, the additional staff time and consumables required (eg. blind sub-culture of bottles, extra incubators for storing bottles and additional QC) to process manual blood cultures should be taken into consideration. Laboratory quality control procedures are an important factor in ensuring accurate results. Internal quality control systems will use media and reagents but the volume will depend on frequency of testing and specific local protocols: these were not included in our analysis and therefore we conservatively estimated the total laboratory cost without the cost of these activities. However, the inclusion of yearly membership for external quality control programs (UK NEQAS in this estimation) was included. Given the variability in local requirements, the costs of referral of isolates for reference laboratory confirmatory testing were not included. We have focused on the three main manufacturers of automated blood culture, ID/AST and MALDI-TOF equipment, however there are other manufacturers and the cost of equipment from these companies may differ substantially. Despite these limitations, this is the first in-depth estimate of the cost of setting up and running a laboratory in Southeast Asia for AMR detection and attempts to cover the main aspects of running a laboratory, using standard equipment from the two dominant microbiology laboratory providers.

In summary, the costs of setting up a microbiology laboratory in a Southeast Asian hospital setting vary significantly by equipment specification and specimen workload. The overall cost of generating data for AMR surveillance was estimated to be between $11–31 per specimen and $105–304 per bacterial isolate. This information should be used by healthcare providers and policy makers to allocate appropriate resources for this global health priority. Unfortunately, this costing exercise indicates that current costs of equipment and consumables for AMR surveillance may be prohibitive in many LMIC settings.

## Supplementary Material

Supporting Information

## Figures and Tables

**Table 1:** Number of specimens processed and requiring antimicrobial susceptibility testing for Laboratory A, B and C in 2019, and the estimated number of samples used in this study for costing calculations. N = number, AST = antimicrobial susceptibility testing, ETT = endotracheal aspirates.

Specimen type		Laboratory A	Laboratory B	Laboratory C	N of specimens used per year in the costing exercise (10,000/100,000)
**Blood culture**	**N**	9089	3518	1460	5000/50000
	**% of specimens**	50	51	61	50
	**N requiring AST**	449	140	50	250/2500
	**% requiring AST**	5	4	3	5
**Urine**	**N**	2317	1783	707	2000/20000
	**% of specimens**	12	26	30	20
	**N requiring AST**	191	105	150	200/2000
	**% requiring AST**	8	6	21	10
**CSF**	**N**	248	388	34	200/2000
	**% of specimens**	1	6	1	2
	**N requiring AST**	22	6	1	10/100
	**% requiring AST**	9	2	3	5
**Sputum/ETT**	**N**	247	38	44	200/2000
	**% of specimens**	1	1	2	2
	**N requiring AST**	86	23	15	100/1000
	**% requiring AST**	35	61	34	50
**Other specimens**	**N**	6336	1136	132	2600/26000
	**% of specimens**	34	17	6	26
	**N requiring AST**	1001	511	64	442/4420
	**% requiring AST**	16	45	48	17
**Total specimens**	**N**	18437	6863	2377	10000/100000
	**N requiring AST**	1759	785	280	1002/10020
	**% requiring AST**	10	11	12	10

**Table 2:** Costs for setting up and running a laboratory in Southeast Asia (USD, 2020) at two capacity levels; a) 10,0000 specimens per year and b) 100,000 specimens per year.

**a) 10,000 specimens per year**	**1. BACTEC no automated AST**	**2. BACTEC and Phoenix**	**3. BACTEC, Phoenix, Bruker MALDI-TOF**	**4. BacT/ALERT no automated AST**	**5. BacT/ALERT and Vitek AST**	**6. BacT/ALERT, Vitek AST, Vitek MS MALDI-TOF**
Total set up cost (1^st^ year)	257,929	336,635	659,700	253,651	291,035	480,115
Total set up cost (5 years)	261,782	340,489	663,554	257,504	294,889	483,969
Total running cost per year	167,151	169,272	175,047	174,067	179,981	213,337
Total running cost (5 years)	835,757	846,360	875,234	870,336	899,906	1,066,683
Average total cost per year	219,508	237,370	307,757	225,568	238,959	310,130
Average cost per specimen per year	21.95	23.74	30.78	22.56	23.90	31.01
Average cost per isolate per year	215.06	232.56	301.52	220.99	234.11	303.84
**b) 100,000 specimens per year**	**1. BACTEC no automated AST**	**2. BACTEC and Phoenix**	**3. BACTEC, Phoenix, Bruker MALDI-TOF**	**4. BacT/ALERT no automated AST**	**5. BacT/ALERT and Vitek AST**	**6. BacT/ALERT, Vitek AST, Vitek MS MALDI-TOF**
Total set up cost (1^st^ year)	402,791	560,123	886,278	394,235	468,519	657,599
Total set up cost (5 years)	416,612	573,945	900,100	408,056	482,340	671,420
Total running cost per year	988,298	961,205	972,335	1,075,348	1,086,767	1,107,976
Total running cost (5 years)	4,941,488	4,806,025	4,861,673	5,376,741	5,433,833	5,539,882
Average total cost per year	1,071,620	1,075,994	1,152,355	1,156,959	1,183,235	1,242,260
Average cost per specimen per year	10.72	10.76	11.52	11.57	11.83	12.42
Average cost per isolate per year	104.99	105.42	112.90	113.35	115.92	121.71

## Data Availability

All data is available within the tables in the publication and the supplemental materials.
