# Supplementary material for: Antimicrobial resistance detection in Southeast Asian hospitals is critically important from both patient and societal perspectives, but what is its cost?
Source: PLOS Glob Public Health. 2021 Oct 13;1(10):e0000018. doi: 10.1371/journal.pgph.0000018 (PMC7611947; doi:10.1371/journal.pgph.0000018)
Supplement: S1 Table — ID = identification, AST = antimicrobial susceptibility testing, AMR = Antimicrobial resistance. (DOCX) [file pgph.0000018.s001.docx]

**S1 Table. List of assumptions used to calculate the overall amount of consumables and reagents required for processing 10,000 specimens.** ID= identification, AST= antimicrobial susceptibility testing, AMR= Antimicrobial resistance

| Costing area | Assumption and calculation | Likely impact |
| --- | --- | --- |
| Conventional AST | For 10,000 specimens approximately 1,002 specimens will need ID and AST. 40% require second line or repeat testing. 1,002 specimens with one Mueller Hinton plate and six antibiotics, 400 with a second Mueller Hinton plate and six antibiotics. 1,500 Mueller Hinton plates allowing for extras and repeats. 1,500 Mueller Hinton plates and 1,500 x 6 antibiotics | Under- or over- estimation depending on the AMR patterns and levels in each country |
| Conventional ID | To simplify and keep in line with the aim of AMR surveillance, the same amount of isolates for ID and AST have been used, however, more ID would probably be completed in a laboratory then AST | Underestimating economic cost if more ID is tested in the laboratory then approximated. Overestimating economic cost if there is less AST completed in the laboratory than ID. |
| Automated AST | For 10,000 specimens approximately 700 will have automated ID/AST (Phoenix or Vitek 2) as this is not done on everything due to simple ID procedures that are often used instead and antibiotic panels which are not covered by the automated AST | Under- or over- estimation depending on independent laboratory practices |
| MALDI-TOF ID | For 10,000 specimens approximately 1,002 isolates require ID by MALDI-TOF. MALDI-TOF isolate spots done in duplicate. 2,004 isolate spots | Under- or over- estimation depending on independent laboratory practices |
| Blood culture | Only one aerobic blood culture bottle costed for. Though generally recommended to do a set of blood cultures, it is common in LMIC settings to do only an aerobic bottle. Setting up of anaerobic system not included in costs | Underestimating economic cost |
| Consumables and reagents | If the amount needed for a consumable is less than the amount in the box/bottle/kit, then a whole box/bottle/kit is costed for the overall cost of running a laboratory as the whole box/bottle/kit would have to be purchased | Overestimating economic cost |
| Currency conversion | Costs predominantly quoted in Thai Baht. Used current conversion of 31.16 Baht= $1 | Change in conversion during the study and afterwards will have different results |
