# Supplementary material for: Antimicrobial resistance detection in Southeast Asian hospitals is critically important from both patient and societal perspectives, but what is its cost?
Source: PLOS Glob Public Health. 2021 Oct 13;1(10):e0000018. doi: 10.1371/journal.pgph.0000018 (PMC7611947; doi:10.1371/journal.pgph.0000018)
Supplement: S2 Table — Fleming report based on a single Kenyan laboratory. Description of what was included to test for each specimen was not included in the Fleming fund report. Costs do not include equipment and staff costs. Currency conversion- $1 = 31.16 Baht, $1 = 0.76 GBD (www.xe.com and https://www.bankofengland.co.uk/ as of 11th August 2020). (DOCX) [file pgph.0000018.s002.docx]

**S2 Table.** **Approximate costs of processing each specimen type including individual consumable and reagent item costs from this analysis compared to the cost from the Fleming laboratory capacity report**. Fleming report based on a single Kenyan laboratory [1]. Description of what was included to test for each specimen was not included in the Fleming fund report. Costs do not include equipment and staff costs. Currency conversion- $1= 31.16 Baht, $1= 0.76 GBD ([www.xe.com](http://www.xe.com) and <https://www.bankofengland.co.uk/> as of 11^th^ August 2020)

| **Specimen type** | **Media and test** | **Quantity** | **Individual cost $** | **Total**  **$** | **Cost from Kenyan laboratory capacity report [1] $** |
| --- | --- | --- | --- | --- | --- |
| **Blood culture** | Blood culture bottle (BACTEC) | 1 | 2.57 | **2.57** |  |
| **Positive Blood culture** | Blood culture bottle (BACTEC) | 1 | 2.57 | 2.57 |  |
|  | Venting needle | 1 | 0.69 | 0.69 |  |
|  | Slide | 1 | 0.02 | 0.02 |  |
|  | Gram stain | 1 | 0.45 | 0.45 |  |
|  | Blood agar (Columbia) | 1 | 0.18 | 0.18 |  |
|  | Chocolate agar | 1 | 0.69 | 0.69 |  |
|  | MacConkey agar | 1 | 0.30 | 0.30 |  |
|  | Plastic petri dish | 3 | 0.12 | 0.36 |  |
|  | **Total** |  |  | **5.26** | **5.07** |
| **CSF** | Universal container | 3 | 0.09 | 0.27 |  |
|  | Slide | 1 | 0.02 | 0.02 |  |
|  | Giemsa stain | 1 | 0.45 | 0.45 |  |
|  | Toluidine blue | 1 | 0.04 | 0.04 |  |
|  | Blood agar (Columbia) | 1 | 0.18 | 0.18 |  |
|  | Chocolate agar | 1 | 0.69 | 0.69 |  |
|  | Sabouraud agar | 1 | 0.13 | 0.13 |  |
|  | Plastic petri dish | 3 | 0.12 | 0.36 |  |
|  | **Total** |  |  | **2.14** | **1.22** |
| **Sputum** | Universal container | 1 | 0.09 | 0.09 |  |
|  | Gram stain | 1 | 0.45 | 0.45 |  |
|  | Slide | 1 | 0.02 | 0.02 |  |
|  | Sputasol | 1 | 0.35 | 0.35 |  |
|  | Centrifuge tube | 1 | 0.29 | 0.29 |  |
|  | Blood agar (Columbia) | 1 | 0.18 | 0.18 |  |
|  | Chocolate agar | 1 | 0.69 | 0.69 |  |
|  | MacConkey agar | 1 | 0.30 | 0.30 |  |
|  | Optochin | 1 | 0.05 | 0.05 |  |
|  | Bacitracin | 1 | 0.32 | 0.32 |  |
|  | Plastic petri dish | 3 | 0.12 | 0.36 |  |
|  | **Total** |  |  | **3.10** | **6.95** |
| **Urine** | Universal container | 1 | 0.09 | 0.09 |  |
|  | Plastic loop | 1 | 0.08 | 0.08 |  |
|  | Chromogenic agar (e.g. Oxoid Brilliance UTI) | 1 | 1.36 | 1.36 |  |
|  | Plastic petri dish | 1 | 0.12 | 0.12 |  |
|  | **Total** |  |  | **1.65** | **2.30** |

1. Seale AC, Hutchison C, Fernandes S, Stoesser N, Kelly H, Lowe B, et al. Supporting surveillance capacity for antimicrobial resistance: Laboratory capacity strengthening for drug resistant infections in low and middle income countries. Wellcome open research. 2017;2.
