# Supplementary material for: Antimicrobial resistance detection in Southeast Asian hospitals is critically important from both patient and societal perspectives, but what is its cost?
Source: PLOS Glob Public Health. 2021 Oct 13;1(10):e0000018. doi: 10.1371/journal.pgph.0000018 (PMC7611947; doi:10.1371/journal.pgph.0000018)
Supplement: S3 Table — (DOCX) [file pgph.0000018.s003.docx]

**S3 Table.** **Approximate reagent and consumable costs for conventional identification and antimicrobial susceptibility testing for key organisms**

| **Organism** | **ID test** | **Quantity** | **Individual cost $** | **Total $** |
| --- | --- | --- | --- | --- |
| ***S. pneumoniae*** | Gram stain | 1 | 0.45 | 0.45 |
|  | Catalase | 1 | 0.34 | 0.34 |
|  | Blood agar (Columbia) | 1 | 0.18 | 0.18 |
|  | Bile esculin solubility | 1 | 0.75 | 0.75 |
|  | Blood Mueller Hinton agar | 2 | 0.48 | 0.96 |
|  | Antibiotic discs | 6 | 0.08 | 0.48 |
|  | E-test (PG, TX) | 2 | 4.81 | 9.62 |
|  | Plastic petri dish | 4 | 0.12 | 0.48 |
|  | **Total** |  |  | **13.26** |
| ***S. aureus*** | Gram stain | 1 | 0.45 | 0.45 |
|  | Blood agar (Columbia) | 1 | 0.18 | 0.18 |
|  | Tube coagulase | 1 | 0.67 | 0.67 |
|  | Staphaurex test | 1 | 2.06 | 2.06 |
|  | DNase agar (1/4 plate) | 1 | 0.10 | 0.10 |
|  | Mueller Hinton agar | 2 | 0.15 | 0.30 |
|  | Antibiotic discs | 6 | 0.08 | 0.48 |
|  | E-test (VA) | 1 | 4.81 | 4.81 |
|  | Plastic petri dish | 4 | 0.12 | 0.48 |
|  | **Total** |  |  | **9.53** |
| ***Salmonella* species** | Gram stain | 1 | 0.45 | 0.45 |
|  | Blood agar (Columbia) | 1 | 0.18 | 0.18 |
|  | Serotyping | 1 | 12.23 | 12.23 |
|  | API 20E (does not include API reagents) | 1 | 5.77 | 5.77 |
|  | Mueller Hinton agar | 1 | 0.15 | 0.15 |
|  | Antibiotic discs | 6 | 0.08 | 0.48 |
|  | Plastic petri dish | 2 | 0.12 | 0.24 |
|  | **Total** |  |  | **19.50** |
| ***E. coli* / *K. pneumoniae*** | Gram stain | 1 | 0.45 | 0.45 |
|  | Blood agar (Columbia) | 1 | 0.18 | 0.18 |
|  | API 20E (does not include API reagents) | 1 | 5.77 | 5.77 |
|  | Mueller Hinton agar | 2 | 0.15 | 0.30 |
|  | Antibiotic discs | 6 | 0.08 | 0.48 |
|  | ESBL confirmation testing discs | 4 | 0.08 | 0.32 |
|  | Plastic petri dish | 3 | 0.12 | 0.36 |
|  | **Total** |  |  | **7.86** |
| ***A. baumannii*** | Gram stain | 1 | 0.45 | 0.45 |
|  | Blood agar (Columbia) | 1 | 0.18 | 0.18 |
|  | Oxidase | 1 | 1.79 | 1.79 |
|  | API 20NE (does not include API reagents) | 1 | 10.71 | 10.71 |
|  | Mueller Hinton agar | 1 | 0.15 | 0.15 |
|  | Antibiotic discs | 6 | 0.08 | 0.48 |
|  | Plastic petri dish | 2 | 0.12 | 0.24 |
|  | **Total** |  |  | **14.00** |
